# INTRINSIC HEALTH: TOWARD A FRAMEWORK FOR THE OBJECTIVE STUDY OF THE BIOLOGY OF HEALTH

**DOI:** 10.1093/geroni/igad104.0114

**Published:** 2023-12-21

**Authors:** Alan Cohen, Martin Picard, Karen Bandeen-Roche

## Abstract

Many definitions of health have been proposed over the years, but all share the characteristic of being subjective and therefore hard to tie to objective measures of biology. While there are undoubtedly subjective aspects of health, here we propose the notion of “intrinsic health” as the capacity of an organism to maintain its internal, biological dynamic equilibrium. Building on first principles, Cohen and Picard show how intrinsic health emerges from the dynamic interplay of energy, communication, and structure at multiple levels of biological organization. The following three talks then propose novel ways to measure intrinsic health. Khan and Wei show how quantile aberrance – modeling when biomarker values are extreme based on the joint distributions of many biomarkers – can be used to quantify intrinsic health. Liu, Xu, Honfo, and Cohen show how metrics of proteome dynamics reflect communication within the organism and can be extracted to quantify health. Finally, Wang and Pei show how transfer entropy – the synchronization between multiple time series – can be extracted to measure health. While these three metrics are still in preliminary stages of development, they show the potential to measure health based on the multivariate dynamics of our internal systems. Going forward, these and other approaches may be integrated to derive robust, objective metrics of intrinsic health, and could serve as the basis for a novel understanding of how aging emerges from declines in intrinsic health over time.

